# Attendance in a national screening program for diabetic retinopathy: a population-based study of 205,970 patients

**DOI:** 10.1007/s00592-022-01946-4

**Published:** 2022-08-12

**Authors:** Anne Suhr Thykjær, N. Andersen, T. Bek, S. Heegaard, J. Hajari, C. S. Laugesen, S. Möller, F. N. Pedersen, L. Rosengaard, K. C. Schielke, R. Kawasaki, K. Højlund, K. H. Rubin, L. Stokholm, J. Grauslund

**Affiliations:** 1 Department of Ophthalmology, Odense University Hospital, J. B. Winsløws Vej 4, Odense C, 5000 Odense, Denmark; 2 Department of Clinical Research, University of Southern Denmark, Odense, Denmark; 3 Steno Diabetes Center Odense, Odense University Hospital, Odense, Denmark; 4 Organization of Danish Practicing Ophthalmologists, Copenhagen, Denmark; 5 Department of Ophthalmology, Aarhus University Hospital, Aarhus, Denmark; 6 Department of Ophthalmology, Rigshospitalet-Glostrup, Copenhagen, Denmark; 7 Department of Ophthalmology, Zealand University Hospital Roskilde, Roskilde, Denmark; 8 Department of Vision Informatics, University of Osaka, Osaka, Japan; 9 Research Unit OPEN, Department of Clinical Research, University of Southern Denmark, Odense, Denmark; 10 Department of Ophthalmology, Aalborg University Hospital, Aalborg, Denmark

**Keywords:** Complications, Diabetes, Diabetic retinopathy, Attendance, Progression, Screening

## Abstract

**Aims:**

A nationwide diabetic retinopathy (DR) screening program has been established in Denmark since 2013. We aimed to perform an evaluation of adherence to DR screenings and to examine whether non-adherence was correlated to DR progression.

**Methods:**

The population consisted of a register-based cohort, who participated in the screening program from 2013 to 2018. We analyzed age, gender, marital status, DR level (International Clinical DR severity scale, none, mild-, moderate-, severe non-proliferative DR (NPDR) and proliferative DR (PDR)), comorbidities and socioeconomic factors. The attendance pattern of patients was grouped as either timely (no delays > 33%), delayed (delays > 33%) or one-time attendance (unexplained).

**Results:**

We included 205,970 patients with 591,136 screenings. Rates of timely, delayed and one-time attendance were 53.0%, 35.5% and 11.5%, respectively. DR level at baseline was associated with delays (mild-, moderate-, severe NPDR and PDR) and one-time attendance (moderate-, severe NPDR and PDR) with relative risk ratios (RRR) of 1.68, 2.27, 3.14, 2.44 and 1.18, 2.07, 1.26, respectively (*P* < 0.05). Delays at previous screenings were associated with progression to severe NPDR or PDR (hazard ratio (HR) 2.27, 6.25 and 12.84 for 1, 2 and 3+ delays, respectively). Any given delay doubled the risk of progression (HR 2.28).

**Conclusions:**

In a national cohort of 205,970 patients, almost half of the patients attended DR screening later than scheduled or dropped out after first screening episode. This was, in particular, true for patients with any levels of DR at baseline. DR progression in patients with delayed attendance, increased with the number of missed appointments.

## Introduction

Diabetic retinopathy (DR) is a frequent complication of diabetes, and sight-threatening DR is among the leading causes of preventable blindness in the working-age population [1]. According to the International Diabetes Federation, the global prevalence of diabetes is 10.5% equivalent to 536.6 million people [2] and amongst patients with diabetes the prevalence of DR is approximately 30% [1]. DR, especially at more severe levels, can have vast physical and emotional consequences for the affected patients, and management of the disease requires many resources from healthcare systems [3]. Diabetic eye screening is a crucial part of disease management for all patients with diabetes. In Denmark, screening is recommended immediately after diagnosis of type 2 diabetes and within five years of diagnosis of type 1 diabetes (at age 12 at the earliest) and lifelong screening is recommended [4]. Non-attendance or delay of scheduled screenings might result in new and potential sight-threatening DR changes, that are not discovered timely, hence delaying proper treatment [5]. Incidence of DR can rise significantly in association with delay of screenings [6]. Still the cause for non- and delayed attendance seems to be multifaceted and optimal attendance might be dependent on both incentives and obstacles being prioritized [7, 8]. No studies have, to our knowledge, examined attendance patterns and the potential health consequences in a population-based cohort.

Denmark has a national tax-funded screening program for DR. It is recommended that patients attend screening at either a practicing ophthalmologist or a hospital-based screening facility. Financial reimbursement is provided regardless of screening site and patients with proliferative DR (PDR) or diabetic macular edema are referred for treatment at the public hospital departments of ophthalmology. Denmark is divided geographically into five regions; the Capital Region of Denmark, Central Denmark Region, North Denmark Region, Region Zealand and Region of Southern Denmark [9]. The regions are responsible for the Danish hospitals and the health services provided by practicing physicians, including practicing ophthalmologists. The capital of Denmark, Copenhagen, is located in the Capital Region of Denmark. Screening is done by either retinal fundus photographs alone or by a combination of photographs and clinical examination. Individualized intervals are planned according to national guidelines [4] and defined by the level of DR as well as glycemic control.

In this study, we aimed to utilize the Danish registers to examine attendance patterns in the Danish nationwide DR screening program, to characterize timely, delayed and one-time attending patients, as well as explore the effects of delayed attendance on DR progression.

## Methods and materials

### Participants

In this retrospective nationwide cohort study, our population was defined by the data in The Danish Registry of Diabetic Retinopathy (DiaBase), which contains data of all patients who had attended DR screening at least once, from January 2, 2013, to December 30, 2018 [10]. We included data from all 591,136 screening visits by 205,970 patients (Table 1), above 18 years of age.

**Table 1:** Characteristics of patients at baseline, according to attendance group

	All, *n* = 205,970	Timely attendance, *n* = 109,135	Delayed attendance, *n* = 73,242	One-time attendance, *n* = 23,593	*P* value
Sex, % Male	116,534 (56.6)	62,567 (57.3)	40,610 (55.4)	13,357 (56.6)	< 0.001
Age, Years (IQR)	66 (55;73)	66 (56;73)	65 (54;73)	66 (54;74)	< 0.001
Marital status					< 0.001
Never married	30,904 (15.0)	16,035 (14.7)	11,050 (15.1)	3819 (16.2)	
Married	118,764 (57.7)	63,833 (58.5)	42,718 (58.3)	12,213 (51.8)	
Widowed or divorced	56,302 (27.3)	29,267 (26.8)	19,474 (26.6)	7561 (32.0)	
Diabetes type, *N* (%)					< 0.001
Type 1 diabetes	16,999 (8.3)	7492 (6.9)	8375 (11.4)	1132 (4.8)	
Type 2 diabetes	153,238 (74.4)	85,786 (78.6)	48,791 (66.6)	18,661 (79.1)	
Unknown	35,733 (17.3)	15,857 (14.5)	16,076 (21.9)	3800 (16.1)	
DR level (ICDR), *N* (%)^A^					< 0.001
No DR	171,633 (83.3)	95,507 (87.5)	55,464 (75.7)	20,662 (87.6)	
Mild NPDR	20,964 (10.2)	9009 (8.3)	10,157 (13.9)	1798 (7.6)	
Moderate NPDR	6551 (3.2)	2405 (2.2)	3583 (4.9)	563 (2.4)	
Severe NPDR	1153 (0.6)	327 (0.3)	687 (0.9)	139 (0.6)	
PDR	5165 (2.5)	1727 (1.6)	3007 (4.1)	431 (1.8)	
Charlson comorbidity index score, *N* (%)^B^					< 0.001
Low	148,615 (72.2)	79,792 (73.1)	51,920 (70.9)	16,903 (71.6)	
Moderate low	27,728 (13.5)	12,984 (11.9)	11,798 (16.1)	2946 (12.5)	
Moderate high	18,721 (9.1)	10,252 (9.4)	6137 (8.4)	2332 (9.9)	
High	10,906 (5.3)	6107 (5.6)	3387 (4.6)	1412 (6.0)	
Screening facility, *N* (%)					< 0.001
Private practice	161,418 (78.4)	89,210 (81.7)	53,241 (72.7)	18,967 (80.4)	
Hospital	44,552 (21.6)	19,925 (18.3)	20,001 (27.3)	4626 (19.6)	
Region of screening, *N* (%)					< 0.001
Capital region of Denmark	53,303 (25.9)	24,363 (22.3)	20,908 (28.5)	8032 (34.0)	
Region Zealand	33,299 (16.2)	17,531 (16.1)	11,332 (15.5)	4436 (18.8)	
Central Denmark region	41,499 (20.1)	24,581 (22.5)	12,733 (17.4)	4185 (17.7)	
North Denmark Region	22,248 (10.8)	9945 (9.1)	9761 (13.3)	2542 (10.8)	
Region of Southern Denmark	55,575 (27.0)	32,690 (30.0)	18,488 (25.2)	4397 (18.6)	
Socioeconomic status					
Income (household net worth), *N* (%)					< 0.001
Low	50,484 (24.5)	23,942 (21.9)	19,704 (26.9)	6838 (29.0)	
Moderate low	50,310 (24.4)	26,383 (24.2)	18,140 (24.8)	5787 (24.5)	
Moderate high	50,953 (24.7)	27,857 (25.5)	17,640 (24.1)	5456 (23.1)	
High	52,660 (25.6)	29,491 (27.0)	17,711 (24.2)	5458 (23.1)	
Education, *N* (%)					< 0.001
Lower secondary	77,796 (37.8)	40,620 (37.2)	27,676 (37.8)	9500 (40.3)	
Upper secondary	85,012 (41.3)	45,902 (42.1)	29,880 (40.8)	9230 (39.1)	
Post-secondary	36,122 (17.5)	19,103 (17.5)	13,232 (18.1)	3787 (16.1)	
Occupation, *N* (%)					< 0.001
Employed or employer	58,533 (28.4)	31,016 (28.4)	21,332 (29.1)	6185 (26.2)	
Student or other	5179 (2.5)	2571 (2.4)	1953 (2.7)	655 (2.8)	
Early retirement	28,404 (13.8)	14,608 (13.4)	10,526 (14.4)	3270 (13.9)	
Retirement	101,135 (49.1)	54,663 (50.1)	34,882 (47.6)	11,590 (49.1)	
Unemployed	12,715 (6.2)	6274 (5.7)	4549 (6.2)	1892 (8.0)	
Ethnic background, *N* (%)					< 0.001
Danish heritage	183,476 (89.1)	98,237 (90.0)	65,072 (88.8)	20,167 (85.5)	
Other heritage	22,457 (10.9)	10,882 (10.0)	8160 (11.1)	3415 (14.5)	

Results given as number (%) or median (IQR). ^A^Classification of DR given by the International Clinical Diabetic Retinopathy Severity Scale [30], ^B^Excluding diabetes

### Data Sources

We utilized the Danish national registers where all data can be linked on an individualized level. This includes entire medical records, socioeconomic data and prescription medication usage.

Diabase, which defined our population, contains data reported by the screening ophthalmologist, and the database has approximately 100,000 additions annually [11]. From DiaBase, we extracted reported and planned screening dates, DR level according to the International Clinical DR severity scale (ICDR scale, no DR = 0, mild non-proliferative DR (NPDR) = 1, moderate NPDR = 2, severe NPDR = 3 or PDR = 4), screening facility (hospital or practicing ophthalmologists) and geographical region of screening (Capital Region of Denmark, Central Denmark Region, North Denmark Region, Region Zealand and Region of Southern Denmark).

In addition to DiaBase, we utilized the following registers:

The Danish Civil Registry (1968) was used to link data across registries using an individual identification number (CPR number) given to all citizens in Denmark [12]. We extracted date of birth, sex (female or male), status (alive, institutionalized, living in Greenland, living abroad, missing or dead) and marital status (never married, married or divorced/widowed). The Danish National Patient Register (1976) contains information on all patients treated at Danish hospitals. This includes the specific department, diagnoses according to the International Classification of Disease (ICD) version ten codes, surgical procedures, treatments and other procedures [13]. The Danish National Prescription Registry (1994) is a unique pharmacological register and one of the largest of its kind worldwide [14]. The registry contains information on all collected prescriptions of medicine nationwide, connected to CPR number. This includes information on the Anatomical Therapeutic Chemical (ATC) classification of the medication as well as detailed information on all prescriptions. The Danish National Patient Register and The Danish National Prescription Registry were utilized for categorizing diabetes type (type 1, type 2 or unknown) [15] as well as to categorize patients' comorbidities according to the Charlson Comorbidity Index score (CCI, 1 = low, 2 = moderate low, 3 = moderate high or 4 = high) [16]. Furthermore, socioeconomic data were acquired from Statistics Denmark [17]; we extracted information on equivalent household income (low, moderate low, moderate high and high), highest achieved level of education (lower secondary, upper secondary and post-secondary) in accordance with the International Standard Classification of Education (ISCED) [18], affiliation to the labor market (employed, student, unemployed, early retirement or retirement) [19] and ethnicity (Danish or other).

### Quantitative variables

**Fig. 1:**
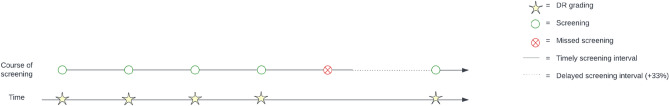
Timeline illustration of intervals and screening visits with DR gradings, indicating risk time as delayed screening interval

The index date was defined as the first screening date, and delay was calculated according to the next recommended screening interval, as given by the screening physician. Patients were classified as having timely attendance if they were never delayed > 33% and did not miss any screenings during follow-up. Patients were classified as delayed if the actual date of the next screening was registered beyond 33% of the intended interval, e.g., a patient with a recommended interval of 90 days, would therefore be classified as delayed if the next screening date was more than 30 days after the planned screening date (Fig. 1).

One-time attendance was defined as a patient only participating in screening once, with no follow-up appointments, without apparent reason. Patients with a scheduled next screening date beyond the observation period or who were referred for treatment (for DR or other eye-related illness) and therefore exited the screening program, as well as patients, who disappeared or died before their next screening, were censored at exit date and, thus, only included in the analyses in the periods where they could be clearly classified. DR progression was defined as a worsening in DR to either severe NPDR or PDR in either eye.

### Statistical methods

Descriptive data on the population were reported in numerical format with percentages for all variables except age, which was reported in median and interquartile range. Statistical significance was calculated using the Chi^2^ test. Using a multinomial logistic regression model with relative risk ratio (RRR) calculations, we compared the characteristics of patients with delayed and one-time attendance to patients with timely attendance depending on various exposure variables. The model included a crude, semi-adjusted (age and gender) and fully adjusted multivariable analysis, adjusted for all statistically significant exposure variables from Table 1 (age, gender, marital status, diabetes type, DR level, modified CCI score (excluding diabetes), screening facility, geographical region of screening, income, education length, occupation and ethnic descent). A multivariable Cox regression model with hazard ratios (HRs) was performed to examine a potential risk of progression in DR level that could be associated with delayed screening intervals. Time-varying analyses were utilized to examine each individual screening period. A period was defined as the time from one screening to next screening and could be timely or delayed. Risk time only included delayed periods, and time splitting at missed screening visits was utilized to define delayed periods from timely periods. A patient stopped contributing with risk time, when they attending a screening again, but could contribute again later on, if another > 33% delay occurred (Fig. 1). All analyses were done in Stata 17 (StataCorp, College Station, Texas, USA), and *P*-values < 0.05 and confidence intervals (CIs) not including 1.0 were considered statistically significant.

## Results

### Descriptive data

**Fig. 2:**
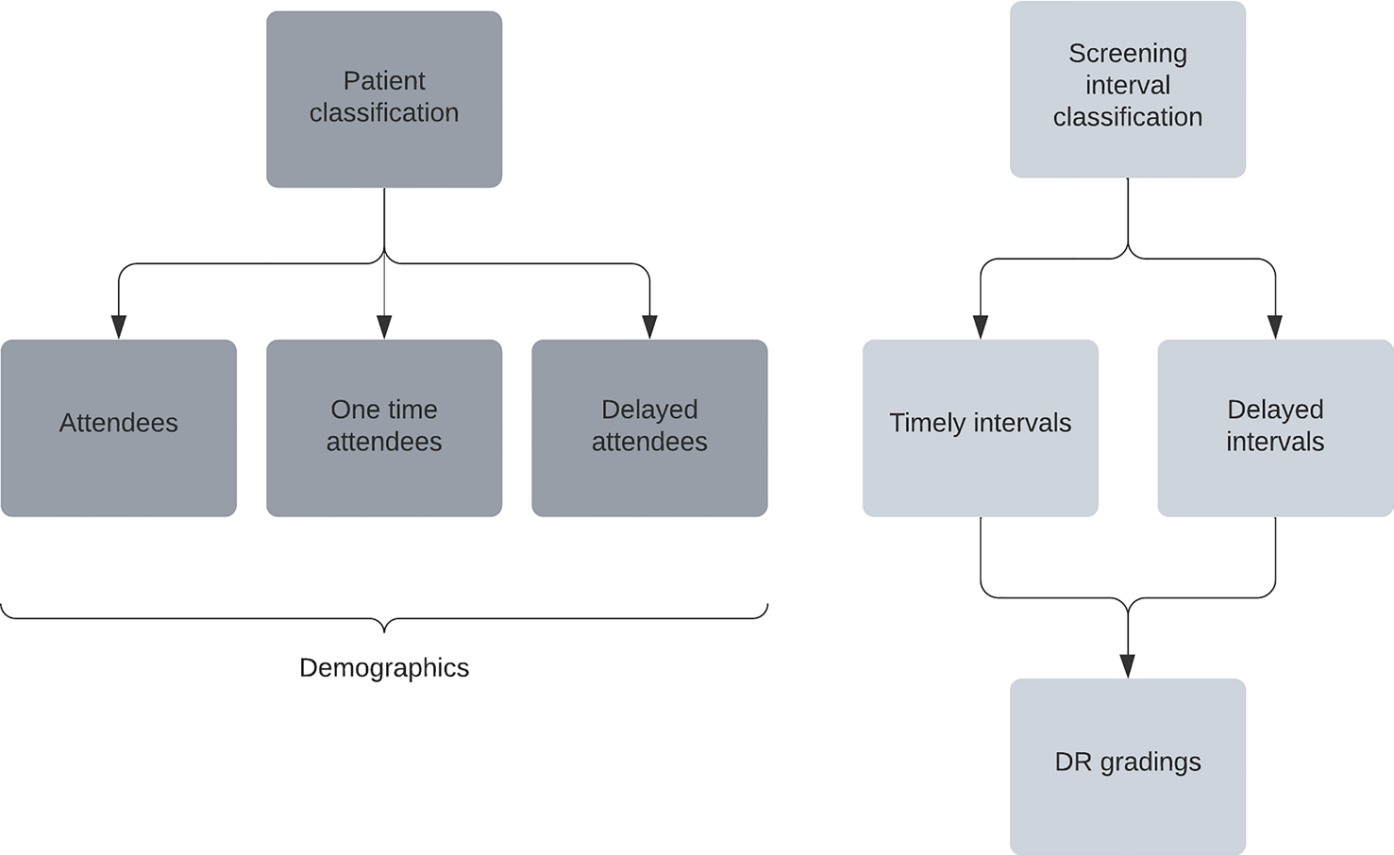
Flowchart with key elements of study design

The population (*n* = 205,970) consisted of 56.6% males, had a median age of 65.7 years (55;73), and 89.1% were of Danish lineage (Table 1, Fig. 2). Baseline prevalence of DR was 16.5% (10.2%, 3.2%, 0.6% and 2.5% for levels 1–4, respectively). Rates of timely attendance, delayed attendance and one-time attendance in the population was 53.0%, 35.5% and 11.5%, respectively. Compared to patients with timely attendance, delayed attendance and one-time attendance were more often observed in females (42.7% vs. 44.6% and 43.4%), non-married patients (14.7% vs. 15.1% and 16.2%) and patients of other ethnic descent than Danish (10.0% vs. 11.1% and 14.5%). Furthermore, compared to patients with timely attendance, patients with delayed attendance had a higher prevalence of DR (12.5% vs. 24.3%), more often type 1 diabetes (6.9% vs. 11.4%) and were screened more frequently at hospitals (18.3% vs. 27.3%). Patients with one-time attendance were more comparable to patients with timely attendance in all three parameters (12.4%, 4.8% and 19.6%). Patients from all five Danish regions were represented, but with varying degrees of adherence. The Central Denmark Region had the highest percentage of attendance, and the North Denmark Region had the lowest (59.2% vs. 44.7%). The North Denmark Region had the highest number of patients with delayed attendance, while the Central Denmark Region had the lowest, within their screened populations (43.9% vs. 30.7%). The highest number of patients with one-time attendance was found in the Capital Region of Denmark and the lowest in the Region of Southern Denmark (15.1% vs. 7.9%). Compared to patients with timely attendance, delayed and one-time attendance were more often observed in patients with lower income (26.9% and 29.0% vs. 21.9%), lower educational level (37.8% and 40.3% vs. 37.2%) and a higher rate of unemployment (6.2% and 8.0% vs. 5.7%).

### Main results

#### Delayed attendance

The multivariable multinomial logistic regression (Table 2) showed that patients with delayed attendance were less likely to be of male gender (RRR 0.94 (95% CI 0.92–0.96)), be older in age (40–59 years (RRR 0.79 (95% CI 0.75;0.85)), 60–79 years (0.76 (95% CI 0.72;0.81)), 80+ years (0.78 (95% CI 0.73;0.84)) and have type 2 diabetes (0.67 (95% CI 0.64;0.70)) compared to patients attending screening at recommended intervals. Having delayed attendance was associated with being either divorced/widowed or married (RRR 1.14 (95% CI 1.10;1.18) and 1.19 (95% CI 1.15;1.23)), having DR level 1–4 (RRR 1.68 (95% CI 1.63;1.74), 2.27 (95% CI 2.14;2.40), 3.14 (95% CI 2.72;3.62), 2.44 (95% CI 2.29;2.61)), a CCI score of 1 (RRR 1.08 (95% CI 1.04;1.11)) and being screened at a hospital based facility (RRR 1.07 (95% CI 1.04;1.10)) in either the Capital Region of Denmark of Denmark (RRR 1.31 (95% CI 1.28;1.35)) or the North Denmark Region (RRR 1.52 (95% CI 1.46;1.58)). Socioeconomically, delayed attendance was mainly associated with having a low income (RRR 1.19 (95% CI 1.16;1.23)), but also being employed (RRR 1.22 (95% CI 1.14;1.31)), in retirement (RRR 1.33 (95% CI 1.23;1.44)) or in early retirement (RRR 1.23 (95% CI 1.14;1.33)).

**Table 2:** Multinomial regression showing the risk of delayed and one-time attendance according to exposure variables, reported in relative risk ratios (RRR) with 95% confidence intervals (CI)

	Delayed attendees					One-time attendees				
	*n* (%)	RRR crude (95%CI)	RRR semi adjusted (95%CI)	RRR fully adjusted (95%CI)	*P*-value	*n* (%)	RRR crude (95%CI)	RRR semi adjusted (95%CI)	RRR fully adjusted (95%CI)	*P*-value
Sex										
Male	40,610 (55.4)	0.93 (0.91;0.94)	0.92 (0.90;0.94)	0.94 (0.92;0.96)	0.0000	13,357 (56.6)	0.97 (0.94;1.00)	0.97 (0.94;1.00)	1.04 (1.01;1.08)	0.0083
Female	32,632 (44.6)	Ref	Ref	Ref		10,236 (43.4)	Ref	Ref	Ref	
Age, Years										
18–39	5823 (8.0)	Ref	Ref	Ref		1538 (6.5)	Ref	Ref	Ref	
40–59	21,027 (28.7)	0.69 (0.66;0.72)	0.69 (0.66;0.72)	0.79 (0.75;0.83)	0.0000	7145 (30.3)	0.89 (0.83;0.94)	0.89 (0.83;0.94)	0.70 (0.65;0.75)	< 0.001
60–79	40,223 (54.9)	0.63 (0.61;0.66)	0.64 (0.61;0.66)	0.76 (0.72;0.81)	0.0000	11,865 (50.3)	0.71 (0.67;0.75)	0.71 (0.67;0.75)	0.53 (0.49;0.58)	< 0.001
80+	6169 (8.4)	0.62 (0.59;0.65)	0.62 (0.59;0.65)	0.78 (0.73;0.84)	0.0000	3045 (12.9)	1.16 (1.08;1.24)	1.16 (1.08;1.24)	0.83 (0.75;0.92)	< 0.001
Marital Status										
Never married	11,050 (15.1)	Ref	Ref	Ref		3819 (16.2)	Ref	Ref	Ref	
Married	42,718 (58.3)	0.97 (0.95;1.00)	1.08 (1.05;1.11)	1.19 (1.15;1.23)	0.0000	12,213 (51.8)	0.80 (0.77;0.84)	0.82 (0.78;0.85)	0.85 (0.81;0.89)	< 0.001
Widowed or divorced	19,474 (26.6)	0.97 (0.94;1.00)	1.11 (1.07;1.15)	1.14 (1.10;1.18)	0.0000	7561 (32.0)	1.08 (1.04;1.13)	1.12 (1.06;1.17)	1.09 (1.03;1.15)	0.0014
Diabetes type, *N* (%)										
Type 1 diabetes	8375 (11.4)	Ref	Ref	Ref		1132 (4.8)	Ref	Ref	Ref	
Type 2 diabetes	48,791 (66.6)	0.51 (0.49;0.53)	0.53 (0.51;0.55)	0.67 (0.64;0.70)	0.0000	18,661 (79.1)	1.44 (1.35;1.54)	1.55 (1.44;1.66)	1.47 (1.36;1.59)	< 0.001
Unknown	16,076 (21.9)	0.91 (0.87;0.94)	0.94 (0.91;0.98)	0.99 (0.95;1.04)	0.7542	3800 (16.1)	1.59 (1.48;1.70)	1.69 (1.57;1.82)	1.53 (1.41;1.66)	< 0.001
DR level (ICDR), *N* (%)^A^										
No DR	55,464 (75.7)	Ref	Ref	Ref		20,662 (87.6)	Ref	Ref	Ref	
Mild NPDR	10,157 (13.9)	1.94 (1.88;2.00)	1.92 (1.86;1.98)	1.68 (1.63;1.74)	0.0000	1798 (7.6)	0.92 (0.88;0.97)	0.92 (0.87;0.97)	0.95 (0.90;1.01)	0.0959
Moderate NPDR	3583 (4.9)	2.57 (2.43;2.70)	2.54 (2.41;2.68)	2.27 (2.14;2.40)	0.0000	563 (2.4)	1.08 (0.99;1.19)	1.08 (0.99;1.19)	1.18 (1.06;1.30)	0.0017
Severe NPDR	687 (0.9)	3.62 (3.17;4.13)	3.51 (3.07;4.00)	3.14 (2.72;3.62)	0.0000	139 (0.6)	1.96 (1.61;2.40)	1.96 (1.61;2.39)	2.07 (1.67;2.57)	< 0.001
PDR	3007 (4.1)	3.00 (2.82;3.18)	2.96 (2.79;3.14)	2.44 (2.29;2.61)	0.0000	431 (1.8)	1.15 (1.04;1.28)	1.15 (1.04;1.28)	1.26 (1.13;1.42)	< 0.001
Charlson comorbidity index score, *N* (%)^B^										
Low	51,920 (70.9)	Ref	Ref	Ref		16,903 (71.6)	Ref	Ref	Ref	
Moderate low	11,798 (16.1)	1.40 (1.36;1.44)	1.41 (1.37;1.45)	1.08 (1.04;1.11)	0.0000	2946 (12.5)	1.07 (1.03;1.12)	1.07 (1.03;1.12)	1.04 (0.99;1.09)	0.1280
Moderate high	6137 (8.4)	0.92 (0.89;0.95)	0.96 (0.93;1.00)	0.87 (0.84;0.90)	0.0000	2332 (9.9)	1.07 (1.02;1.13)	1.08 (1.03;1.14)	1.07 (1.02;1.13)	0.0068
High	3387 (4.6)	0.85 (0.82;0.89)	0.91 (0.87;0.95)	0.74 (0.71;0.78)	0.0000	1412 (6.0)	1.09 (1.03;1.16)	1.10 (1.04;1.17)	1.09 (1.02;1.16)	0.0090
Screening facility, *N* (%)										
Practicing Ophthalmologist	53,241 (72.7)	Ref	Ref	Ref		18,967 (80.4)	Ref	Ref	Ref	
Hospital	20,001 (27.3)	1.68 (1.64;1.72)	1.65 (1.61;1.69)	1.07 (1.04;1.10)	0.0000	4626 (19.6)	1.09 (1.05;1.13)	1.09 (1.05;1.14)	0.92 (0.88;0.96)	< 0.001
Region of screening, *N* (%)										
Capital region of Denmark	20,908 (28.6)	1.33 (1.29;1.37)	1.31 (1.28;1.35)	1.30 (1.26;1.34)	0.0000	8032 (34.0)	1.30 (1.25;1.36)	1.30 (1.25;1.36)	1.31 (1.26;1.37)	< 0.001
Region Zealand	11,332 (15.5)	Ref	Ref	Ref		4436 (18.8)	Ref	Ref	Ref	
Central Denmark region	12,733 (17.4)	0.80 (0.78;0.83)	0.79 (0.77;0.82)	0.77 (0.74;0.79)	0.0000	4185 (17.7)	0.67 (0.64;0.70)	0.67 (0.64;0.70)	0.68 (0.65;0.72)	< 0.001
North Denmark region	9761 (13.3)	1.52 (1.46;1.57)	1.52 (1.46;1.57)	1.52 (1.46;1.58)	0.0000	2542 (10.8)	1.01 (0.96;1.07)	1.01 (0.96;1.07)	1.01 (0.96;1.08)	0.6338
Region of Southern Denmark	18,488 (25.2)	0.87 (0.85;0.90)	0.87 (0.85;0.90)	0.84 (0.81;0.87)	0.0000	4397 (18.6)	0.53 (0.51;0.56)	0.53 (0.51;0.56)	0.51 (0.49;0.54)	< 0.001
socioeconomic status										
income (Household net worth), *N* (%)										
Low	19,704 (26.9)	1.20 (1.17;1.23)	1.19 (1.16;1.22)	1.19 (1.16;1.23)	0.0000	6838 (29.0)	1.30 (1.25;1.35)	1.30 (1.25;1.35)	1.18 (1.13;1.24)	< 0.001
Moderate low	18,140 (24.8)	Ref	Ref	Ref		5787 (24.6)	Ref	Ref	Ref	
Moderate high	17,640 (24.1)	0.92 (0.90;0.95)	0.93 (0.90;0.95)	0.91 (0.89;0.94)	0.0000	5456 (23.2)	0.89 (0.86;0.93)	0.89 (0.86;0.93)	0.92 (0.88;0.96)	< 0.001
High	17,711 (24.2)	0.87 (0.85;0.90)	0.88 (0.86;0.90)	0.84 (0.82;0.87)	0.0000	5458 (23.2)	0.84 (0.81;0.88)	0.84 (0.81;0.88)	0.85 (0.81;0.89)	< 0.001
Education, *N* (%)										
Lower secondary	27,676 (39.1)	0.98 (0.96;1.01)	1.01 (0.98;1.04)	0.97 (0.94;1.00)	0.0254	9500 (42.2)	1.18 (1.13;1.23)	1.19 (1.14;1.24)	1.11 (1.06;1.16)	< 0.001
Upper secondary	29,880 (42.2)	0.94 (0.92;0.97)	0.95 (0.93;0.98)	0.94 (0.91;0.96)	0.0000	9230 (41.0)	1.01 (0.97;1.06)	1.02 (0.97;1.06)	0.97 (0.93;1.02)	0.2127
Post-secondary	13,232 (18.7)	Ref	Ref	Ref		3787 (16.8)	Ref	Ref	Ref	
OCCUPATION, *N* (%)										
Employed or employer	21,332 (29.1)	0.91 (0.85;0.96)	1.10 (1.03;1.17)	1.22 (1.14;1.31)	0.0000	6185 (26.2)	0.78 (0.72;0.86)	0.76 (0.69;0.83)	0.93 (0.84;1.03)	0.1658
Student or other	1953 (2.7)	Ref	Ref	Ref		655 (2.8)	Ref	Ref	Ref	
Early retirement	10,526 (14.4)	0.95 (0.89;1.02)	1.10 (1.03;1.19)	1.23 (1.14;1.33)	0.0000	3270 (13.9)	1.18 (1.07;1.31)	1.16 (1.04;1.28)	0.85 (0.76;0.95)	0.0043
Retirement	34,882 (47.6)	0.95 (0.89;1.01)	1.20 (1.12;1.28)	1.33 (1.23;1.44)	0.0000	11,590 (49.1)	0.88 (0.80;0.97)	0.84 (0.76;0.93)	0.87 (0.78;0.98)	0.0209
Unemployed	4549 (6.2)	0.84 (0.79;0.89)	1.30 (1.21;1.40)	1.08 (0.99;1.16)	0.0680	1892 (8.0)	0.83 (0.76;0.91)	0.77 (0.69;0.85)	1.03 (0.92;1.16)	0.5775
Ethnic background, *N* (%)										
Danish heritage	65,072 (88.9)	Ref	Ref	Ref		20,167 (85.5)	Ref	Ref	Ref	
Other heritage	8160 (11.1)	1.13 (1.10;1.17)	1.08 (1.05;1.12)	0.98 (0.94;1.01)	0.2181	3415 (14.5)	1.53 (1.47;1.59)	1.53 (1.47;1.60)	1.20 (1.14;1.26)	< 0.001

Data are given as numbers (*n*, (%)) and relative risk ratios (95% CI). Reference group is patients with timely attendance. ^A^Classification of DR given by the International Clinical Diabetic Retinopathy Severity Scale [30], ^B^Excluding diabetes. Semi adjusted model adjusted for sex and age. Fully adjusted model adjusted for all statistically significant variables in Table 1

#### One-time attendance

One-time attendance was associated with being male (RRR 1.04 (95% CI 1.01;1.08)), divorced or widowed (RRR 1.09 (95% CI 1.03;1.15)), having type 2 diabetes (RRR 1.47 (95% CI 1.36;1.59)), DR level 2–4 ((RRR 1.18 (95% CI 1.06;1.30), 2.07 (95% CI 1.67;2.57), 1.26 (95% CI 1.13;1.42)) or CCI scores of 2 or 3 ((RRR 1.07 (95% CI 1.02;1.13), 1.09 (95% CI 1.02;1.16)) compared to patients attending screening at recommended intervals (Table 2). One-time attending patients were more likely to be screened in the Capital Region of Denmark of Denmark (RRR 1.31 (95% CI 1.26;1.37)). One-time attendance was inversely associated with age (40–59 (RRR 0.70 (95% CI 0.65;0.75)), 60–79 (0.53 (95% CI 0.49;0.58)) and 80+ (0.83 (95% CI 0.75;0.92)). Socioeconomically one-time attendance was associated with a low income (RRR 1.18 (95% CI 1.13;1.24)), lower educational length (RRR 1.11 (95% CI 1.06;1.16)) and other ethnic heritage than Danish (RRR 1.20 (95% CI 1.14;1.26)).

#### Progression

**Fig. 3:**
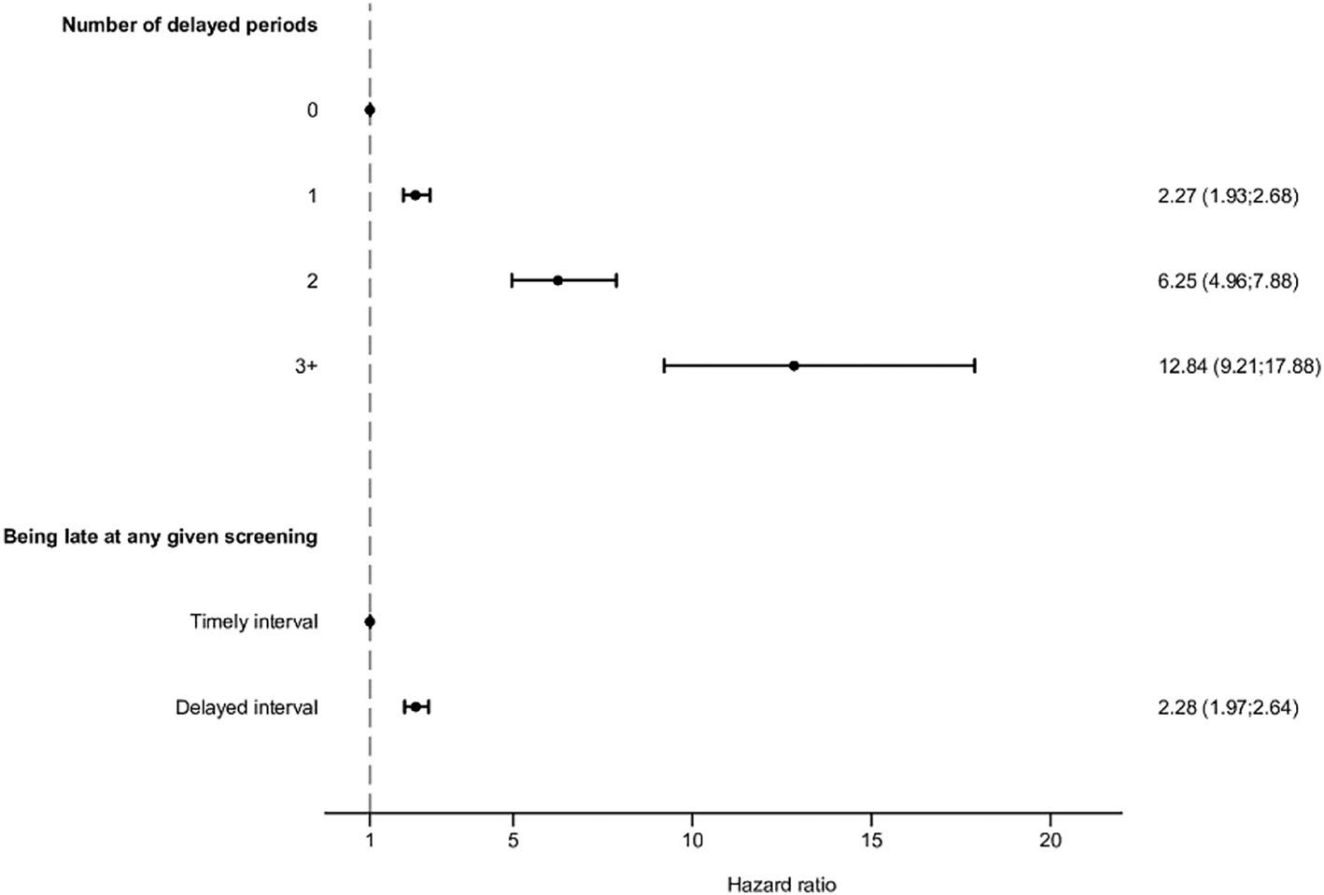
Forest plot illustrating the findings of Table 3; the risk of progression to severe non-proliferative diabetic retinopathy (DR) or proliferative DR (PDR) according to number of delayed periods and at any given screening Data are given in hazard ratios (HRs) with confidence intervals (CI)

Cox regression analysis (Table 3, Fig. 3) showed that any delay in screening resulted in double the risk of progression to severe NPDR or PDR (2.28 HR (95% CI 1.97;2.64). Patients with past delayed intervals were more likely to experience disease progression to severe NPDR or PDR during follow-up; the risk increased by the number of missed appointments so that patients with delays in 1, 2 or 3+ appointments had increased risks of HR 2.27 (95% CI 1.93;2.68), HR 6.25 (95% CI 4.96;7.88) and HR 12.84 (95% CI 9.21;17.88) for progression, compared to patients who attended screenings timely.

**Table 3:** Risk of progression to severe non-proliferative diabetic retinopathy (DR) or proliferative DR according to number of delays, given in hazard ratios (HR) and 95% confidence intervals (CI)

	Events	Risk time	Crude HR (CI 95%)	Semi adjusted HR (CI 95%)	Fully adjusted HR (CI 95%)	*P*-value
Number of delayed periods						
0	1015	324,108.39	Ref	Ref	Ref	
1	670	123,844.96	2.34 (1.97;2.79)	2.26 (1.90;2.68)	2.27 (1.93;2.68)	< 0.001
2	229	12,869.24	8.80 (6.89;11.24)	7.40 (5.82;9.41)	6.25 (4.96;7.88)	< 0.001
3+	75	1812.18	21.15 (15.13;29.57)	17.18 (12.29;24.03)	12.84 (9.21;17.88)	< 0.001
Any given delay						
Timely interval	1321	379,180.86	Ref	Ref	Ref	
Delayed interval	574	80,626.27	2.09 (1.81;2.42)	2.07 (1.79;2.39)	2.28 (1.97;2.64)	< 0.001

Data are given as numbers and hazard ratios (confidence interval). Semi adjusted model adjusted for sex and age. Fully adjusted model adjusted for all statistically significant variables in Table 1. ^A^Progressions. ^B^Risk time given in person-days per 1000

## Discussion

This study is, to our knowledge, the most extensive study in the field of attendance to DR screening, utilizing 591,136 screening episodes by 205,970 patients with diabetes in a nationwide cohort. Our research showed that delayed attendance and one-time attendance of DR screenings were associated with younger age, divorce, lower income, screening in the Capital Region of Denmark, as well as higher levels of DR and competing illnesses. Progression to more advanced DR (severe NPDR and PDR) was seen more often in patients with delayed attendance, and the number of delays was correlated to a significantly increased risk of progression. This is in accordance with a study from England [6], in which the number of missed screenings were examined in a retrospective observational study of 62,067 patients in the North East London Diabetes Eye Screening Programme. A 20% increase in the incidence of referable DR was demonstrated in patients that missed ten or more consecutive appointments. We found that patients age 40 years and above were less likely to be delayed or have one-time attendance, compared to the 60–79 age group. Which is in agreement with previous studies from England [20–23], Ireland [24] and Scotland [25] thus confirming a trend across DR screening programs internationally. Delayed attendance was observed to be vastly increasing according to more severe DR levels at baseline compared to patients with no DR. Paradoxically, the patients who needed the timely screenings the most, were the ones who utilized it the least. This, in turn, could also be part of the explanation as to why their DR was in fact of a more severe level. It should be noted that the groups of patients diagnosed with severe NPDR made up a small percentage of the cohort as a whole, and therefore, there might be a larger statistical uncertainty in the results for these patients. Several studies examining the incentives and barriers of patients to DR screening found that a great facilitator to attendance was the knowledge of the potential consequences of non-attendance on vision and DR progression [24, 26–28]. This could be a point of focus to ensure proper communication and dissemination of DR awareness from healthcare professionals to patients with diabetes—in Denmark, as well as internationally. This could also help combat the anxiety that might counterintuitively keep some patients from attending a screening, because the fear of a severe examination result or the possible societal stigma is too overwhelming. Patients who attended screenings at practicing ophthalmologists were more adherent to their given intervals than patients at hospitals. Because of the centralization of larger hospitals in Denmark, access to practicing ophthalmologists might be logistically easier and more accessible to patients, especially in rural areas. Distance to the screening facility has previously been shown as a barrier to screening [8]. To increase the convenience for patients, DR screenings can often be timed with other diabetes-related screenings including podiatry, cardiology and endocrinology appointments at most Danish hospitals. Attendance in the different geographical regions of Denmark varied; although we observed a greater non-adherence in the North Denmark Region, we also observed this in the Capital Region of Denmark of Denmark, where patients were more likely to have both delayed and one-time attendance. This could be due to the more diverse population composition in metropolitan areas, including younger people, with lower incomes. Technical issues, partly due to the implementation of a new electronic medical record system, might also have affected the data received in DiaBase from hospitals in Region Zealand and the Capital Region of Denmark, introducing a potential bias. Patients with type 2 diabetes were more likely to only attend screening once compared to patients with type 1 diabetes. This could be due to the fact that type 2 diabetes often is discovered later in life, and perhaps in relation to other lifestyle-related illnesses; patients might, therefore, not be accustomed to the sudden burden of appointments this entails. One-time attendance could partly be explained by patients with pre-diabetes or who are undergoing a medical investigation to determine a potential diabetes diagnosis, that have been recommended a screening by their general practicing physician. We found a correlation between both delayed and one-time attendance and general comorbidity in regards to higher CCI scores across the regression analysis, indicating that patients who are suffering from competing illnesses might not have the surplus to also keep up screening at timely intervals, or at all. Socioeconomic deprivation in terms of low income and unemployment was seen as risk factor for delayed and one-time attendance. The risk of non-adherence was lower in patients with higher incomes, showcasing a potential distortion and inequality in health care access according to income. Several studies credit socioeconomic deprivation as the leading cause of non-attendance [25, 29], and even though an association in a Danish setting is apparent in regard to low income and non-Danish descent, it might not be as stark due to the generally flatter societal structure as well as the completely tax-funded healthcare system, where no out of pocket expenses are needed. Length of education did not significantly change the odds of delayed or one-time attendance, as seen in previous studies [7].

The inclusion of a large nationwide cohort with a considerable amount of screenings, and detailed, validated register information on an individualized level, is a clear strength of this study. The addition of socioeconomic data ensured the completeness of the characterization of the study population.

As our study focused on adherence to the screening program, and patients attending DR screening at least once, we did not address the issue of patients never attending screening, which might add another dimension. Due to the register-based nature of the study, the subjective reasons for non-adherence were not addressed. This would, however, be important for future reference, as an involvement of patients and a prioritization of their prerogative will be crucial in order to improve attendance.

In conclusion, our study of non-adherence successfully added information on a population basis using a national cohort of patients in the Danish screening program of DR in Denmark. We highlighted younger age, divorce, presence of DR, competing illnesses and low income as the characteristics of patients with delayed and one-time attendance in the Danish screening program and showed twice the risk of progression to severe NPDR and PDR in patients with delayed attendance.

## Data Availability

The datasets created and analyzed during the current study are available from the Danish Health Data Authority and Statistics Denmark, but restrictions apply to the availability of these data, which were used under license from OPEN and Danish Health Data Authority and are not publicly available.
